# Surgery of the Intestines

**Published:** 1905-08-26

**Authors:** 


### SURGERY OF THE INTESTINES.

The methods of **Intestinal Resection and Anastomosis** are legion, but that we have not reached finality in our methods is shown by the fact that the inventive genius of various surgeons is still being displayed in the description of new methods for effecting these objects. McGraw[1] has brought forward a new operation for intestinal stenosis and points out that the difficulty in dealing with such cases is the risk of infection of the peritoneum and the difference in calibre of the portion of gut above and below the point of obstruction. The German surgeons have sought in various ways to overcome these difficulties. Mikulicz was the first to abandon the "one time" operation and to resort to a method of operating in which the gut at one operation was fastened outside the abdominal cavity, in a second resected, and in a third repaired. Of 12 cases thus treated ten recovered. Hochenegg has had success with a similar operation. He brings the tumour well out of the abdomen, prevents its retraction by passing a fold of iodoform gauze through the mesentery, and closes the abdominal wound around the bowel with sutures. After a few hours he makes a small opening in the distended afferent limb, inserts a glass tube, and empties the intestine. At the end of ten or twelve days the abdomen is reopened, the tumour and adjacent parts of the gut are excised, and the two ends united with circular suture.

McGraw considers the existing methods faulty. He contends that the main and essential things in operations for colonic cancer are: First, the protection of the abdominal cavity from infection by deferring the evacuation of the intestinal contents until the abdominal wound has been completely closed and hermetically sealed. This can be done only by bringing the tumour out of the abdomen and fastening it there while the gut is opened. Second, the avoidance of all secondary operations which would bring new risk to the patient. He proposes to accomplish these objects in the following manner:—Given a tumour or stenosis every effort should be made to locate the seat of the disease, as the knowledge of its location enables the surgeon to make the abdominal incision at the most favourable point. If the tumour can be approximately located the abdomen should be opened directly over it. If that is impossible, the exploratory incision should be made in the median line. The tumour or stricture having been found, the coil in which it is seated should be drawn out of the abdomen, and, if necessary for this purpose, adhesions should be broken or cut, enlarged mesenteric glands should be removed, and the mesentery repaired. At this stage the two limbs of the intestine leading to and from the tumour, at a sufficient distance from the seat of the disease, should be joined together by Lembert sutures and anastomosis made by an elastic ligature. When the wound has been closed and sutured to the protruding intestine, a firm silk ligature should be tied round the efferent limb just below the tumour, and the gut cut off below it, care being taken that not the least portion of its contents is allowed to come in contact with the wound. It may now be closed with a circular suture, inverted into itself, and pushed in until its outer surface no longer projects beyond the level of the skin. A few Lembert sutures will now hold it in position, and the integuments may now be brought over it and sutured. In this way the efferent limb is once and for all disposed of. Layers of gauze soaked in collodion should then be placed over the whole wound so as to seal it hermetically. A large trocar, such as is used for ovarian cysts, may now be thrust into the intestine and the fæces discharged. When the gut has been emptied of fæces, the diseased portion should be cut off and a large glass tube inserted into the bowel and tied in place. At the end of four or five days the fæces will find their way through the new channel of anastomosis and pass into the lower bowel. As soon as this is determined by fæcal discharges per rectum, the tube may be removed, the gut sutured and turned into itself, and the wound in the integuments closed over it. Of mechanical appliances for intestinal anastomosis, one of the latest devices is a soluble button suggested by Paterson.[2] Such a button gets rid of the objection, which obtains in the case of the non-soluble buttons, that a second operation is never necessary for its removal. After many experiments Paterson came to the conclusion that gelatine treated with chrome alum would meet the various requirements and this material has the additional advantage that it can be prepared in such a manner as to be more or less resistent to the action of the digestive juices, according to the activity of these in the part operated upon, or to suit the particular purpose which the surgeon may have in view. By varying the strength of the solution or the time that this is allowed to act, or by both these means, almost any degree of insolubility can be obtained, though the stronger the solutions the more brittle does the gelatine become. He finds that the most suitable strength for intestinal buttons is ten grains of chrome alum, dissolved in one ounce of cold water. The buttons, which are not unlike Murphy's in shape, are made by pouring a strong watery solution of gelatine into moulds of suitable size, these having been previously lined with a layer of strong muslin or dressing cloth. This fibrous material becomes saturated with the gelatine and incorporated with the outer part of the button, and gives additional strength, while at the same time diminishing the tendency to brittleness. When the gelatine has set the casts are removed and are placed in the chrome solution, of which there should be an ounce or so for each button. The length of time they should remain in this depends on the size of the button and also on the degree of insolubility required, but casts up to three-quarters of an inch in diameter should soak for from ten to twelve hours, by which time the solution will have penetrated the whole mass. They are then kept in 50 per cent. alcohol till required. A button three-quarters of an inch in diameter prepared as described resists solution for from five to seven days. The method of fixing this support is practically the same as that employed in using Murphy's buttons. No cases are quoted in which this soluble button has been used, so it has yet to be put to the test of practicability.

**Acquired Diverticula of the Intestine** until recently were regarded as pathological curiosities, but Beer[3] calls attention to the work of Klebs, Hanau, Hausemann, Groser, and others, owing to which their importance in relation to other abdominal conditions is becoming gradually known. According to the old classification, diverticula were divided into false and true. The former became identified with acquired, the latter with congenital. False diverticula were considered mucosal herniæ through the muscularis, their walls consisting of serous, submucosa, and mucosa. True diverticula, on the other hand, were made up of all layers of the bowel wall, and Meckel's diverticulum was practically the only representative of this class. Recent work has shown that diverticula similar in structure to the true may develop, during the patient's lifetime, and as they have nothing to do with an embryonic development of a congenital structure, they should not be called true diverticula. Many acquired diverticula are due to traction, but this is not always the case. Those diverticula of all the layers of the gut wall not due to traction are often called true diverticula, as they differ from the true in no essential detail, as far as their intrinsic structure is concerned. They differ, however, in that they are acquired, and in this respect they and the more common mucosal herniæ above mentioned are grouped together. Both are acquired and both often develop in the same intestine. The acquired diverticula are found in the small intestine, in the appendix, colon, sigmoid flexure, and rectum. At times hundreds have been found in one intestinal tract. It is fairly well agreed that they are most frequently found in the sigmoid, and that, in general, they are much more common than was once thought. As far as their causation is concerned a unanimity of opinion does not exist. It is generally granted that old people alone show them, and especially such old people as have suffered from long-standing constipation. Heschl and others, to find the cause of these diverticula, experimented by filling the intestines of human corpses with water and noting where the injected bowels ruptured. They found that the rupture occurred regularly into the mesentery, and from this they concluded that here was the weakest part of the bowel; consequently just here diverticula developed. It does not, however, follow that this is the weakest part of the living intestinal wall, and therefore it must be conceded that conclusions, based on such experiments, as to the origin of false diverticula must be invalid. No one can deny, after studying acquired diverticula, that the veins and their sheaths have a definite influence on the development of diverticula on the mesenteric side of the gut, but from the fact that these diverticula often develop on the non-mesenteric sides, where no large veins pierce the bowel wall, it is more than likely that those on the mesenteric side do no more than direct the path along which the diverticula develop. It is highly probable that one and the same cause is present, no matter where the diverticula develop, and the fact that diverticula occur in old people, in people whose intestines have been more or less worked out, as evidenced by constipation, points to a muscular deficiency, and in this muscular weakness the cause of the formation of false diverticula is probably to be sought. That diverticula are not harmless is shown by numerous reports, though they less often cause trouble in the small intestine because of the fluid nature of the contents of the small gut. Beer has found six cases in the literature where diverticula produced stenosis of the sigmoid or upper rectum. Some of these cases were associated with so much perisigmoiditis that it was difficult to diagnose them from carcinoma. Sometimes diverticula lead to perforation into the peritoneum and the sigmoid should always be examined in cases of perforative peritonitis when the surgeon is in doubt as to the source of the infection. Diverticula may lead to abscesses or localised peritonitis in the left iliac fossa, and such cases make it clear that we should think of acquired diverticula when we are dealing with inflammatory conditions in the left iliac fossa in elderly people. Diverticula may lead to perforation into the urinary bladder or may become densely adherent to the bladder, and occasionally they are associated with thickening and contraction of the mesentery. It has been suggested that diverticula may be the cause of appendicitis and it is possible that the chronic ulcerations in false diverticula may lead to the development of carcinoma. Hochenegg has related such a case.

[1] Annals of Surgery, Nov. 1904, p. 689. [2] Lancet, April 1, 1905, p. 858. [3] Amer. Jour. Med. Sc., July 1905, p. 135.

